# Chronic Inflammatory Demyelinating Polyneuropathy (CIDP): A Comprehensive Review of Types, Pathophysiology, and Treatment Approaches

**DOI:** 10.1002/brb3.71458

**Published:** 2026-04-29

**Authors:** Ayesha Khan, Arsal Khan, Kuldeep Dalpat Rai, Anzel Saeed, Harsh Kumar, Aneesh Kumar Sangtiani, Tehreem Fatima, Muhammad Tanveer Alam, Faiza Rajput, Sakshi Chawla, Hussain Haider Shah, Humaira Kalam

**Affiliations:** ^1^ Department of Medicine Dow University of Health Sciences Karachi Pakistan; ^2^ Shaheed Tajuddin Ahmad Medical College Gazipur Bangladesh; ^3^ Voice of Doctors Research School Dhaka Bangladesh

**Keywords:** autoimmune disorders, chronic inflammatory demyelinating polyneuropathy, demyelination, efgartigimod, emerging therapies, FcRn blockers, intravenous immunoglobulin, neuropathy treatment, pathophysiology, plasma exchange

## Abstract

**Purpose of Review**: This review aims to provide a comprehensive analysis of chronic inflammatory demyelinating polyneuropathy (CIDP), focusing on its clinical manifestations, pathophysiology, and treatment advancements, with particular emphasis on efgartigimod as a novel therapeutic agent.

**Recent Findings**: CIDP is a chronic autoimmune disorder caused by demyelination and axonal degeneration, leading to progressive weakness and impaired sensory function. While conventional treatments, including corticosteroids, IVIg, and plasma exchange, provide symptomatic relief, their limitations include significant side effects and resistance in some cases. Efgartigimod, an FcRn blocker targeting the IgG catabolic pathway, has emerged as a promising therapy. The ADHERE study demonstrated a 61% reduction in CIDP relapses with efgartigimod, alongside improved disability scores and a favorable safety profile.

**Summary**: CIDP presents significant diagnostic and therapeutic challenges, requiring tailored approaches to management. Efgartigimod introduces a targeted mechanism of action, offering hope for patients unresponsive to traditional therapies. Further research is needed to establish its long‐term efficacy and optimal role in CIDP treatment strategies.

## Graphical abstract

Chronic inflammatory demyelinating polyneuropathy is an autoimmune disorder causing demyelination, leading to distal weakness, sensory loss, and autonomic dysfunction. Immune activation triggers macrophage‐mediated myelin damage and conduction failure. Treatments include steroids, IVIg, plasma exchange, and emerging therapies like efgartigimod, which reduce IgG levels and relapse risk.

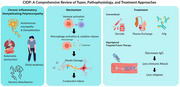

## 1 Introduction

Chronic inflammatory demyelinating polyneuropathy (CIDP) is a chronic autoimmune disorder characterized by progressive muscle weakness and impaired sensory function due to inflammation and damage to the peripheral nerves (Dimachkie and Barohn 2013). It is due to demyelination and axonal degeneration resulting from a complex interplay of immune dysregulation, significantly affecting the patient's quality of life, often leading to impaired mobility, dependence on assistive devices, and psychological distress (Dimachkie and Barohn 2013; Koike and Katsuno 2020).

The prevalence of CIDP is estimated to be approximately 0.7–10.3 cases per 100,000 individuals, with a slightly higher incidence in males than females (Broers et al. 2019). Its widespread presence highlights the need for effective therapeutic options.

Traditional treatments for CIDP primarily focus on immunomodulation to reduce inflammation and nerve damage. These include intravenous immunoglobulin (IVIg), which supplies healthy antibodies, plasma exchange (PE), which therapeutically removes circulating pathogenic autoantibodies, immune complexes, and complement components from the plasma, thereby reducing immune‐mediated damage to peripheral nerves; and corticosteroids, which suppress the immune system (Kuwabara et al. 2017). All of these therapies have demonstrated clinical benefit in randomized controlled trials and are recommended in international treatment guidelines. In addition, subcutaneous immunoglobulin (SCIg) has emerged as an effective maintenance therapy for CIDP, supported by evidence from the phase III PATH randomized controlled trial (Van Schaik et al. 2018).

Rather than acting through a single mechanism, these therapies exert complex immunomodulatory effects. Immunoglobulin therapy (IVIg and SCIg) modulates immune responses through multiple pathways, including inhibition of complement activation, modulation of Fc receptor signaling, and neutralization of pathogenic autoantibodies. PE removes circulating immune mediators such as pathogenic antibodies and complement components, whereas corticosteroids exert broad anti‐inflammatory and immunosuppressive effects that reduce immune‐mediated nerve injury (Caballero‐Ávila et al. 2025).

Other immunosuppressive agents, including azathioprine and cyclophosphamide, have been used in clinical practice, particularly in treatment‐refractory cases; however, their efficacy in CIDP has not been consistently confirmed in controlled clinical trials, and their use is generally considered off‐label or supported by limited evidence (Mahdi‐Rogers et al. 2017).

These treatments offer symptomatic relief and improve the quality of life for many patients. However, they often have significant side effects and may not be effective for everyone. Also, the emergence of treatment‐resistant CIDP cases emphasizes the urgent need for novel therapeutic approaches (Van Den Bergh et al. 2022; Gorson 2012).

In this context, the recent FDA approval of efgartigimod marks a significant milestone in the treatment regimen of CIDP (Proposed Mechanism of Action 2024). Efgartigimod is a neonatal Fc receptor (FcRn) blocker that offers a new mechanism of action by targeting the immunoglobulin G (IgG) catabolic pathway. This innovative approach holds promise for improving patient outcomes by addressing the underlying pathophysiology of CIDP (Allen, Basta, et al. 2024).

This narrative review aims to provide a comprehensive overview of CIDP, including its clinical manifestations, pathophysiology, and effect on the patient's quality of life. Furthermore, it will explore the historical landscape of CIDP treatment, featuring the limitations of existent therapies. It will also highlight the available evidence on efgartigimod. This review will assess its potential as a transformative treatment option for CIDP patients by comparing its efficacy and safety profile to traditional treatment modalities. Ultimately, this review seeks to contribute to a deeper understanding of CIDP and its management.

## 2 Types of CIDP

The 2021 European Academy of Neurology/Peripheral Nerve Society guidelines define CIDP as a heterogeneous spectrum comprising typical CIDP and subtypes (distal, multifocal, focal, motor, and sensory), rather than separate atypical entities. Diagnosis is based on a combination of clinical features and electrodiagnostic evidence of demyelination, supported by findings such as elevated cerebrospinal fluid (CSF) protein or treatment response. Clinically, CIDP presents with a chronic (≥ 8 weeks), progressive or relapsing course, characterized by variable patterns of motor weakness, sensory involvement, and reduced or absent reflexes, with the distribution and modality of deficits distinguishing the subtypes (Van Schaik et al. 2018; Van Den Bergh et al. 2022), which are summarized in Table 1.

**TABLE 1:** Types of CIDP.

Type of CIDP	Clinical criteria	Electrodiagnostic criteria
Typical CIDP	‐ Symmetric proximal and distal weakness in upper and lower limbs ‐ Sensory involvement in ≥ 2 limbs ‐ Absent or reduced reflexes in all limbs ‐ Progressive or relapsing over ≥ 8 weeks	‐ Demyelinating abnormalities in ≥ 2 motor nerves ‐ Sensory abnormalities in ≥ 2 nerves
Distal Acquired Demyelinating Symmetrical Neuropathy (DADS)	‐ Distal sensory loss and weakness, especially in lower limbs ‐ Gait instability	‐ Demyelinating abnormalities in ≥ 2 upper limb motor nerves (CMAP ≥ 1 mV) ‐ Sensory abnormalities in ≥ 2 nerves
Multifocal CIDP	‐ Asymmetric, multifocal sensory, and motor deficits ‐ Often upper limb predominant	‐ Motor abnormalities in ≥ 2 nerves across more than one limb ‐ Sensory abnormalities in ≥ 2 affected limb nerves
Focal CIDP	‐ Weakness and/or sensory loss in a single limb or localized nerve territory	‐ Motor abnormalities in ≥ 2 nerves in the same limb ‐ Sensory abnormality in ≥ 1 nerve in the same limb
Motor CIDP	‐ Symmetric proximal and distal motor weakness only ‐ No clinical sensory symptoms	‐ Motor demyelinating abnormalities in ≥ 2 nerves ‐ Normal sensory conduction in ≥ 4 nerves (median, ulnar, radial, sural)
Sensory CIDP	‐ Sensory ataxia, vibration, and position sense loss ‐ No motor weakness	‐ Sensory conduction abnormalities in ≥ 2 nerves ‐ Normal motor conduction in ≥ 4 nerves (median, ulnar, peroneal, tibial)

### 2.1 Typical CIDP

As the name suggests, these patients typically present with symmetrical, progressive, or relapsing weakness affecting both proximal and distal muscles, alongside sensory deficits like numbness and tingling. Symptoms usually develop over at least two months and involve both motor and sensory nerves, often leading to greater proximal muscle weakness and difficulties in activities such as climbing stairs. Electrophysiological findings include prolonged distal latency, reduced conduction velocities, conduction block, and temporal dispersion, with nerve conduction studies (NCS) indicating demyelination. CIDP generally responds well to immunotherapy, including corticosteroids, IVIg, and plasmapheresis, resulting in significant symptom improvement for most patients (Lewis 2017).

### 2.2 Multifocal Acquired Demyelinating Sensory and Motor

The asymmetric variant of CIDP also referred to as “Lewis–Sumner syndrome” (L‐SS) or Multifocal Acquired Demyelinating Sensory and Motor (MADSAM), encompasses asymmetrical involvement of the upper and/or lower extremities. This variant is characterized by pain, motor conduction blocks in nerves, raised protein levels in CSF and elevated anti‐GM1 antibody titres in rare instances (Rajabally and Chavada 2009). Patients who receive treatment show a good response with IVIgs. Although steroids are also an option for treatment, they are found to be less effective than IVIgs (Viala et al. 2004).

### 2.3 Pure Motor CIDP

This type mainly affects the muscles responsible for movement. Patients typically experience progressive or relapsing weakness, particularly in the proximal muscles of their arms and legs, while sensory issues are usually minimal. Electrophysiological tests often show signs of demyelination in the motor nerves, such as prolonged distal latency, reduced conduction velocities, conduction block, and temporal dispersion. This demyelination can lead to difficulties with tasks that require fine motor skills, like wrist drop or foot drop. Fortunately, most patients respond well to treatments like IVIg and corticosteroids, with significant improvements in muscle strength, especially when therapy begins early. However, some individuals may not respond as effectively to corticosteroids, indicating a need for further exploration of alternative treatment options (Sabatelli et al. 2001).

### 2.4 Pure Sensory CIDP

Clinically, patients typically present with sensory symptoms, including numbness, tingling, and pain in the distal limbs, while motor function usually remains intact, exhibiting minimal to no weakness. Electrophysiological assessments reveal demyelination in sensory nerves, characterized by prolonged distal latencies and reduced sensory nerve action potentials (SNAPs), along with possible conduction block. Treatment responses vary; many patients show improvement with immunotherapy, such as corticosteroids or IVIg, resulting in diminished sensory symptoms and enhanced quality of life (Menon et al. 2021).

### 2.5 Focal CIDP

It is caused by localized demyelination in peripheral nerves, leading to asymmetrical weakness or sensory deficits in specific areas, as opposed to the generalized symptoms seen in classic CIDP. Electrophysiological assessments typically reveal localized conduction blocks and reduced conduction velocities in affected nerve segments, necessitating targeted NCS to avoid misdiagnosis. Patients generally respond positively to immunotherapy, including corticosteroids and IVIg (Rizzuto et al. 1998).

### 2.6 Distal Acquired Demyelinating Symmetric Neuropathy

Distal Acquired Demyelinating Symmetrical Neuropathy (DADS) is characterized by sensory perceptual symptoms in the lower limbs, without affecting adjacent limbs or cranial nerves. Patients typically report numbness, tingling, and challenges with fine motor skills, while their proximal muscle strength usually remains intact. Electrophysiological evaluations demonstrate demyelination features such as prolonged distal latencies, decreased conduction velocities, and evidence of conduction block, primarily impacting distal motor nerves. DADS is often linked to paraprotein‐related neurodegenerative disorders, with IgM paraproteinemia commonly associated with this condition. It can be divided into idiopathic DADS (DADS‐I) and DADS with amplified monoclonal protein (DADS‐M), with approximately 50%–70% of DADS‐M patients testing positive for anti‐myelin‐associated glycoprotein (MAG) antibodies. This suggests a unique pathophysiology and treatment response that differs from CIDP. DADS typically responds favorably to immunotherapy options such as corticosteroids, IVIg, and plasmapheresis (Menon et al. 2021).

## 3 Morphological Characteristics

Inflammatory lesions in CIDP are primarily located in the spinal roots, proximal nerve trunks, and major plexuses, although they can also be dispersed throughout the peripheral nervous system (PNS). Since it is difficult to directly access the proximal nerves and nerve roots, biopsies were performed on the sural nerve, which revealed a wide range of pathological changes. These changes include oedema, demyelination, and the formation of onion bulbs, axonal degeneration, and inflammatory infiltrates of macrophages and T cells that may be found in perivascular or in endoneurial regions (Prineas and McLeod 1976; Sommer et al. 2005; Bosboom et al. 1999; Pollard et al. 1986).

### 3.1 Pathogenesis of CIDP

**FIGURE 1:**
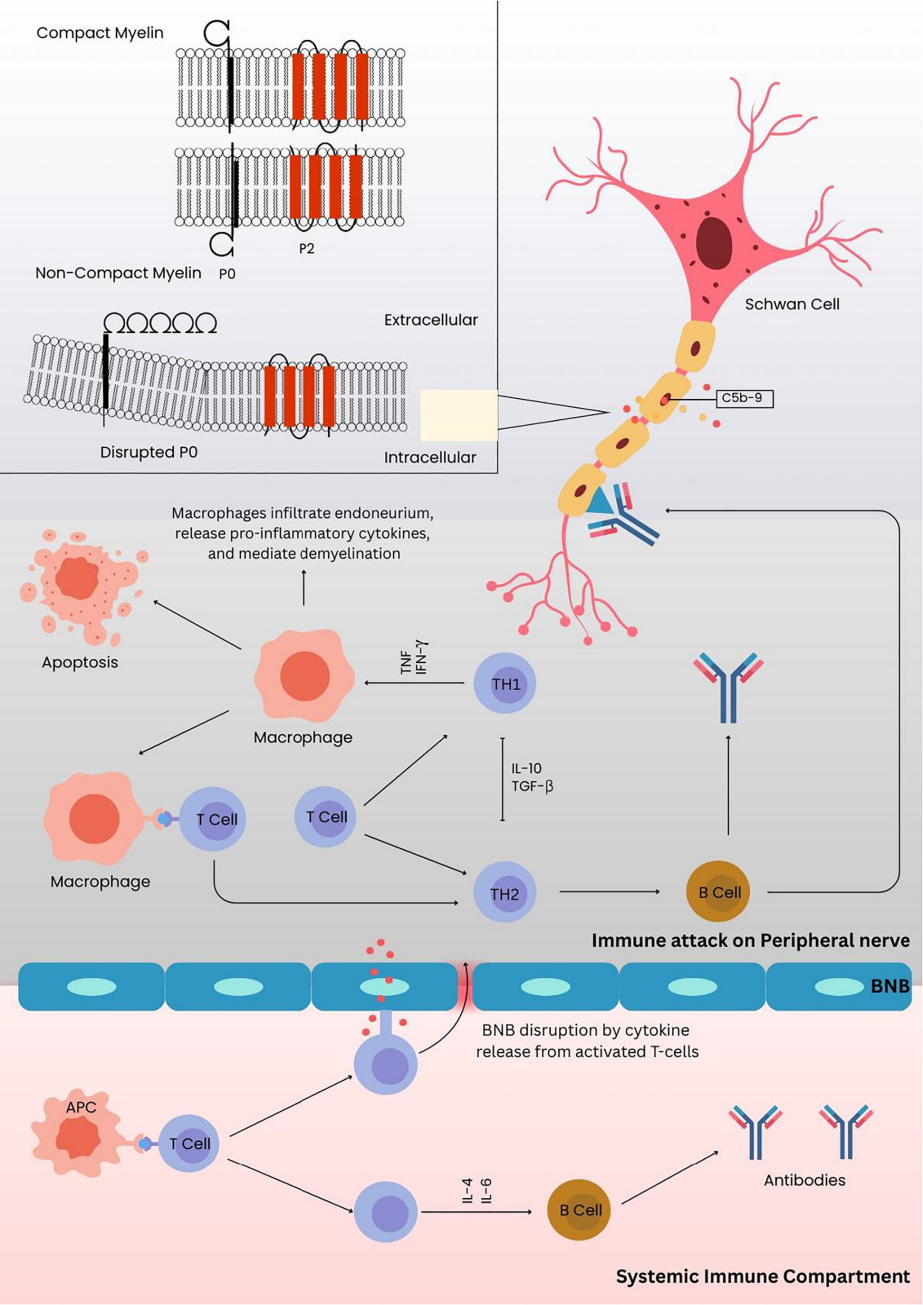
Pathogenesis of CIDP. The figure illustrates three key stages: Peripheral immune activation, blood–nerve barrier disruption, nerve infiltration, and demyelination. Macrophages are shown contributing to demyelination and release of pro‐inflammatory cytokines along with T‐cell activation and apoptosis. The binding of autoantibodies to myelin or nodal structures can further disrupt nerve conduction and contribute to axonal dysfunction. Arrows indicate the migration and interactions of immune cells and the flow of pathogenic factors.

The pathogenesis of CIDP is immune‐mediated, involving components from both cell‐mediated and humoral immunity, resulting in damage to peripheral nerves. There are several lines of evidence supporting CIDP as an autoimmune reaction against undefined Schwann cell and myelin antigens. First, effective treatment of the disease using drugs that target the immune system, including IVIg, PE, and corticosteroids, strongly supports an autoimmune etiology. In addition, evidence of an inflammatory response, involving humoral and cell‐mediated immunity, in the blood and peripheral nerves, also favors an autoimmune‐mediated pathogenesis. Although some patients reported infections before the onset of neurological symptoms, no target or trigger of the autoimmune response linked to an infectious etiology could be identified; hence, no infectious agent has been definitively associated with disease pathogenesis. The overall immune‐mediated mechanisms contributing to nerve damage in CIDP are illustrated in Figure 1.

### 3.2 Cellular Mechanisms

Cellular immune mechanisms are believed to play a crucial role in the pathogenesis of CIDP. This is supported by several lines of evidence, including the presence of inflammatory infiltrates in sural nerve biopsies, the expression of cytokines, alterations in the frequencies and functions of T cell subsets, and the detection of inflammatory mediators in the blood and CSF of CIDP patients (Schmidt et al. 1996; Hartung et al. 1991; Rentzos et al. 2012; Gironi et al. 2010; Chi et al. 2010; Madia et al. 2009; Press et al. 2003).

#### 3.2.1 Disruption of Blood–Nerve Barrier

A pivotal precursor to nerve inflammation and subsequent damage in CIDP is the disruption of the blood–nerve barrier (BNB). Physiologically, BNB maintains endoneurial homeostasis by restricting the movement of soluble factors, such as serum proteins, from the blood into the neural environment. However, when T cells are activated, they cross the BNB and modulate its permeability, allowing the entry of normally restricted molecules.

During the active disease phase, CD4+ T cells in the peripheral circulation secrete proinflammatory cytokines, including IL‐2, interferon‐gamma (IFN‐γ), and interleukin‐17 (IL‐17), along with chemokines such as interferon gamma‐induced protein 10 (IP‐10) and macrophage inflammatory protein 3β (MIP‐3β) (Hartung et al. 1991; Madia et al. 2009; Press et al. 2003). In addition, CD4+ T cells mediated upregulation of activation markers such as T‐bet and pSTAT1 (Madia et al. 2009) is also observed, which may result in further activation of macrophages and upregulation of adhesion molecules, such as vascular cell adhesion molecule‐1 (VCAM‐1) (Archelos et al. 1999), endothelial leukocyte adhesion molecule‐1 (ELAM‐1) (Oka et al. 1994), and intercellular adhesion molecule‐1 (ICAM‐1) (Musso et al. 1994), on the endothelium of vessels in proximity to the nerves.

Activated T cells adhere to these endothelial cells via adhesion molecules, roll along the vascular surface, and migrate across the BNB. During this process, proinflammatory cytokines and chemokines (Hartung et al. 1991) and enzymes such as matrix metalloproteinases are secreted by T cells, further degrading BNB and exacerbating the immune response within the nerve. Upon visualization using gadolinium‐enhanced magnetic resonance imaging (MRI) of nerve trunks or plexuses in patients with CIDP (Kuwabara et al. 1997), degradation of BNB can be observed, a critical process that allows soluble factors, such as antibodies, to access the endoneurium.

#### 3.2.2 Inflammatory Cell Infiltration

Biopsies of the sural nerve in patients with CIDP show the infiltration of different types of inflammatory cells, including CD4+ T cells, CD8+ T cells (Schneider‐Hohendorf et al. 2012), and macrophages (Schmidt et al. 1996). The upregulation of major histocompatibility complex class II (MHC II) molecules (Pollard et al. 1986) along with B7‐1 and B7‐2 (Kiefer et al. 2000; Murata and Dalakas 2000). Infiltrating macrophages and Schwann cells help re‐activate local T cells. Various types of cells within the nerve express inflammatory cytokines like TNFα, IFNγ, and IL‐2, increasing the immune response (Mathey et al. 1999).

Macrophages are the primary infiltrating inflammatory cells and tend to cluster around endoneurial vessels (Sommer et al. 2005). Both resident and recruited macrophages are frequently observed in CIDP nerve biopsies and are thought to contribute to the immune response by presenting antigens and releasing pro‐inflammatory cytokines or toxic mediators. They also play a crucial role in the later phases of demyelination, where they participate in the removal and engulfment of myelin (Kiefer et al. 2001). Ultrastructural analysis of CIDP nerve biopsies reveals macrophages infiltrating between the spirals of Schwann cell plasma membranes, including the outer mesaxon, causing the breakdown of myelin lamellae by extending elongated processes within the lamellae (Vital et al. 2000).

#### 3.2.3 The Role of CD8± T Cells

A notable increase in MHC class I molecules has been observed in Schwann cells of CIDP‐affected nerves, potentially aiding in the activation of cytotoxic CD8+ T cells. A specific foreign or self‐antigen has not been identified as a CD8+ target in CIDP; however, clonal expansion of CD8+ T cells has been observed in sural nerve biopsies and peripheral blood (Schneider‐Hohendorf et al. 2012), suggesting that these CD8+ T cell clones are concentrated in the nerve. This indicates that CIDP pathogenesis can be explained as an antigen‐mediated, CD8+ cell–dependent attack.

Recent studies of the T cell profile in CIDP individuals have revealed increased activation of CD8+ T cells relative to CD4+ T cells, which decreases after IVIg therapy (Mausberg et al. 2013). This oligoclonal activation of CD8+ T cells is commonly seen as a sign of a T cell response to chronic infection, even though no specific infectious agent has consistently been linked to CIDP.

#### 3.2.4 Role of Regulatory T Cells and Central Tolerance

In CIDP, there is evidence of dysregulation of the immune response, regulating excessive or inappropriate immune activation (Sanvito et al. 2009; Chi et al. 2008). More specifically, there is a decrease in the number of circulating Tregs identified by CD4+ CD25high Foxp3+ markers (Chi et al. 2008), and these isolated cells show a reduced ability to suppress proliferative reactions when compared to Tregs from healthy people (Sanvito et al. 2009; Chi et al. 2008). This imbalance in the Treg compartment could contribute to the immune dysfunction observed in CIDP.

### 3.3 Humoral Mechanisms

#### 3.3.1 Autoantibody Responses to Major Myelin Proteins

The importance of humoral mechanisms in the pathogenesis of CIDP is highlighted by the effectiveness of PE in its treatment. Moreover, indirect evidence from biopsy and serological studies reinforces the role of humoral immune responses. Immunoglobulins and complement components are seen deposited on Schwann cells' outer surface and the compact myelin in sural nerve biopsies (Dalakas and Engel 1980) from certain CIDP patients. Indirect immunofluorescence has demonstrated the binding of serum from some CIDP patients to normal nerve sections (Yan et al. 2000). Significantly, among a group of patients who had a good reaction to PE, the sera that attached to nerve sections led to demyelination and a decrease in conduction speed (Yan et al. 2000) when injected into rats intramurally. Further examination revealed that compact myelin protein P0 is a target antigen (Yan et al. 2001). However, the specific autoantigen attacked by autoantibodies in the majority of CIDP patients is still unknown.

#### 3.3.2 Autoantibodies Against Nodal Regions of Myelinated Axons

Research on autoantibody specificity has now shifted from major myelin proteins to those in non‐compact myelin regions, especially in CIDP and selected forms of Guillain–Barré Syndrome (GBS). These consist of the Ranvier node, paranode, and juxtaparanode (Man et al. 2012; Devaux et al. 2012; Devaux 2012).

Cell adhesion molecules (CAMs) like gliomedin, NrCAM, and NF186 play a crucial role in both the early formation (Amor et al. 2014) and ongoing maintenance (Amor et al. 2014) of Na+ channel clustering at the node of Ranvier. Axoglial junctions at the adjacent paranode involve contacting‐1/caspr‐1 complexes that bind to Schwann cell neurofascin 155 (Salzer et al. 2008), thereby connecting paranodal loops to the axonal membrane. NF155 plays a crucial role in the segregation of ion channels, the formation of the paranodal structure, and the facilitation of efficient nerve conduction (Thaxton et al. 2010).

Recent studies have identified autoantibodies directed against nodal and paranodal proteins such as neurofascin‐155, neurofascin‐186, contactin‐1, and Caspr1 (Liu et al. 2022; Cortese et al. 2019; Johnson et al. 2025; Broers et al. 2024). However, neuropathies associated with these antibodies are increasingly recognized as autoimmune nodopathies or paranodopathies rather than classical CIDP (Liu et al. 2022; Broers et al. 2024). Therefore, while these findings provide important insights into immune‐mediated mechanisms affecting nodal structures, they represent distinct disease entities and should be interpreted separately from typical CIDP pathogenesis.

Nevertheless, the study of these nodal and paranodal proteins has improved understanding of how immune‐mediated disruption of axoglial junctions can impair saltatory conduction and contribute to conduction failure in immune‐mediated neuropathies (Cortese et al. 2019; Johnson et al. 2025).

#### 3.3.3 Diagnostic Dilemmas and Crossover Phenomenon in CIDP and Related Disorders

The diagnosis of CIDP is a challenging process due to its heterogeneous clinical manifestations, overlap with other neuropathic conditions, and absence of specific biomarkers. Although diagnostic accuracy has improved after the introduction of European Academy of Neurology/European Academy of Neurology (EAN/PNS) guidelines, some critical issues remain (Van Den Bergh et al. 2022; Thakkar et al. 2024).

These challenges included diagnosing atypical forms of CIDP (distal, multifocal, focal, motor, and sensory forms) that might not meet the electrodiagnostic criteria for typical CIDP variants. Some of these variants may switch from atypical to typical form, further blurring the diagnostic criteria. Another diagnostic issue is acute‐onset CIDP (A‐CIDP), which may be confused with GBS (Van Den Bergh et al. 2022; Thakkar et al. 2024).

Electrodiagnostic testing is one of the key aspects of the diagnosis of CIDP, but upto 20% cases do not meet the electrophysiological criteria (Van Den Bergh et al. 2022; Thakkar et al. 2024). In these cases, we have to depend on CSF studies, nerve imaging, nerve biopsy, or treatment response for diagnosis (Van Den Bergh et al. 2022; Thakkar et al. 2024). These investigations have their own limitations, an increase in CSF protein is non‐specific, MRI and ultrasound have variable sensitivity and inter‐rater reliability, and nerve biopsy is invasive and has low sensitivity (Van Den Bergh et al. 2022; Thakkar et al. 2024).

Further complexities can arise from autoimmune nodopathies being diagnosed as CIDP. The antibodies in these patients are directed to paranodal and nodal structures (e.g, neurofascin‐155, contactin‐1, and Caspr1) and demonstrate poor response to standard CIDP therapies (Van Den Bergh et al. 2022). These conditions may have distinct clinical and pathological features and require different diagnostic and treatment modalities (Van Den Bergh et al. 2022; Thakkar et al. 2024).

Besides, the clinical presentation of CIDP might be distorted by the presence of some comorbid conditions, including diabetes mellitus, monoclonal gammopathy of undetermined significance (MGUS), HIV infection, or a systemic malignancy (Van Den Bergh et al. 2022). Collectively, these issues prompt the relevance of an integrative diagnostic process. Special care should be taken to prevent underdiagnosis as well as overdiagnosis of CIDP. Misdiagnosis of mimicking conditions can expose the patient to immunotherapy with no clinical effects (Van Den Bergh et al. 2022).

## 4 Investigations

The diagnosis can be achieved through various methods, including clinical assessment, neuroimaging, ELISA, triple stimulation technique (TST), NCS, and CSF analysis. The diagnostic criteria are listed in Table 1.

### 4.1 Laboratory and Immunologic Findings

CSF analysis typically shows elevated protein levels without an increase in cell count, a finding known as **albuminocytologic dissociation**. This indicates disruption of the BNB due to inflammatory processes affecting peripheral nerves. Pleocytosis may occur and is associated with an increased presence of CD123+ dendritic cells. This elevation of CD123+ dendritic cells indicates a potential role for these immune cells in the inflammatory processes. Some patients may also present with autoantibodies targeting peripheral nerve components, suggesting an immune response against the myelin sheath (Illes and Blaabjerg 2017).

### 4.2 Nerve Conduction Studies

NCS in CIDP patients typically reveal several characteristic findings, including prolonged distal latency, reduced conduction velocities, and recurrent prolongation of F‐response latency, all indicative of demyelination. In addition, NCS may demonstrate complete or partial conduction block, as evidenced by decreased amplitude of the compound muscle action potential (CMAP) when comparing proximal stimulation to distal stimulation (Kamm 2016). Temporal dispersion may also be present, characterized by the spreading of action potential waveforms, where signals from different motor units arrive at slightly different times. These findings indicate variable and patchy patterns of distribution of lesions (Dyck and Tracy 2018; Towman and Hamdan 2020). Commonly affected nerves include the peroneal, tibial, ulnar, and median nerves, which may show both demyelination and axonal damage (Cortese et al. 2016).

### 4.3 Immunological Testing

Autoantibodies can be detected by enzyme‐linked immunosorbent assay (ELISA), cell‐based assays, and immunofluorescence (Mathey et al. 2017). These may include anti‐ganglioside antibodies (targeting GM1, GD1a, and other gangliosides), and anti‐Contactin‐1 (CNTN1) antibodies; neurofascin (NF155 and NF186) (Rajabally and Chavada 2009; Querol and Illa 2015). These autoantibodies were previously associated with CIDP but are now classified as a distinct disorder named autoimmune nodopathies (Van Den Bergh et al. 2022). These tests now have limited diagnostic utility for CIDP but can prove useful in distinguishing between CIDP and autoimmune nodopathies (Rajabally and Chavada 2009; Querol and Illa 2015).

### 4.4 Neuroimaging Techniques

Nerve ultrasound typically reveals enlarged cross‐sectional areas of peripheral nerves and can differentiate between disease phases in treatment‐naïve patients. Diffusion tensor imaging (DTI) findings show significant reductions in values for the upper and lower extremities, particularly in the ulnar and sciatic nerves, when compared to control subjects. In addition, studies have demonstrated increased dimensions of ganglia and nerve roots in CIDP patients, with lumbar plexus root diameters nearly twice those of healthy controls (Lehmann et al. 2019).

In conclusion, a comprehensive approach that integrates patient history with detailed diagnostic methods, including electrodiagnostic and laboratory assessments, enables clinicians to achieve a precise diagnosis, differentiate between types of CIDP, and distinguish it from other neuropathic disorders. This approach not only supports targeted treatment and management tailored specifically to CIDP, but also ensures that therapeutic strategies align with the unique characteristics of the condition, ultimately improving patient outcomes and treatment effectiveness.

## 5 Acute Manifestation Of CIDP

CIDP is usually chronic and reaches nadir after 8 weeks, but in about 16% of the cases of CIDP, it has been observed that patients had rapidly progressive disease and reached nadir within 8 weeks (Mansour et al. 2020). This acute manifestation of CIDP is known as A‐CIDP. Many symptoms of A‐CIDP overlap with another demyelinating condition known as acute inflammatory demyelinating polyneuropathy, which is a subtype of GBS (Yadavaraj 2023). It is important to differentiate A‐CIDP from GBS, especially in cases of GBS that exhibit treatment‐related fluctuations or GBS‐TRF (deterioration in GBS symptoms after partial initial improvement). A‐CIDP can share features with GBS‐TRF in early phases of the disease. Many articles and studies have been published to clarify this distinction, as the treatment and prognosis of these two conditions vary significantly (Sung et al. 2014). One of the distinguishing features is that the course of A‐CIDP persists or relapses after 8 weeks, which is unlikely for GBS. Furthermore, GBS‐TRF is typically treated with multiple courses of IVIg or PE, whereas A‐CIDP is treated with long‐term maintenance therapy that includes corticosteroids, IVIg, and PE. The electrophysiological studies of A‐CIDP show similar trends to CIDP, representing demyelinating changes (Inan et al. 2024).

## 6 Prognosis

Studies revealed a variable prognosis in patients with CIDP. In one study, 26% of patients had complete remission requiring no treatment for two years, and 61% had a partial response to treatment (Niu et al. 2024; Kuwabara et al. 2006). However, the overall prognosis of CIDP could not be generalized due to multiple subtypes of CIDP, time of presentation, severity, and response to treatment (Niu et al. 2024). It has been observed that patients who were more clinically impaired and who had MADSAM were more likely to relapse (Niu et al. 2024). While the risk of relapse was lower in pure motor and distal CIDP as compared to typical CIDP. The poor prognosis was also related to a delay in treatment, revealing that greater latency between treatment and symptom presentation was associated with a more severe disability in the long term, revealing that initial disability in the disease might not be treated with later therapy (Al‐Zuhairy and Jakobsen 2023). The mode of progression, acute or chronic, is also an important prognostic factor, translating to response to treatment and refractoriness. Prognosis is favorable, with very few patients having severe morbidities (6%) and mortality in long‐term CIDP (1%) (Niu et al. 2024; Al‐Zuhairy and Jakobsen 2023; Al‐Zuhairy et al. 2020).

## 7 Differential Diagnosis for CIDP

Multiple conditions mimic CIDP, and careful clinical and laboratory evaluation is essential to identify subtle distinctions that aid in early and accurate diagnosis of CIDP and improve the prognosis of the disease before it has a profound impact on an individual's life. These conditions include:

(a) Guillain–Barré Syndrome (GBS), (b) Multifocal Motor Neuropathy (MMN), (c) Hereditary Neuropathies (CMT1A), (d) POEMS Syndrome, (e) AL Amyloidosis, (f) Vasculitic Neuropathy, and (g) Autoimmune Nodopathies (anti‐NF155, CNTN1, CASPR1).

The features that overlap between these conditions and CIDP are described below in Table 2 and the distinguishing features, diagnostic clues, and treatment response differences are summarized in Table 3.

**TABLE 2:** Overlapping features of different conditions with CIDP.

Condition	Overlapping features with CIDP
**Guillain–Barré syndrome (GBS)**	1. Demyelinating neuropathy with areflexia, symmetric weakness. 2. Sensory involvement, such as paresthesia or ataxia 3. Elevated proteins in CSF 4. Autoimmune etiology 5. IVIg and plasma exchange are commonly used to treat GBS.
**Multifocal motor neuropathy (MMN)**	1. Demyelinating neuropathy is debatable 2. Nerve conduction block 3. Progressive motor weakness 4. Respond to IVIg
**Charcot–Marie–Tooth disorders** **(CMTDs)**	1. Distal weakness occurs in both 2. Slowly progressive demyelination. 3. Absent reflexes
**POEMS syndrome**	1. Demyelinating neuropathy 2. Elevated CSF protein 3. Progressive course 4. Symmetrical polyneuropathy
**Amyloid neuropathy**	1. Involvement of both sensory and motor nerves. 2. Areflexia, symmetric weakness. 3. Elevated CSF proteins may be present. 4. Progressive course
**Vasculitic neuropathy**	1. Peripheral nerve involvement 2. Weakness and sensory loss with areflexia 3. Nerve conduction abnormalities 4. Elevated inflammatory markers
**Paraneoplastic neuropathy**	1. Demyelination is rare but can occur 2. Elevated CSF proteins may be present 3. Progressive course 4. Weakness and sensory loss with areflexia in some types 5. Abnormal nerve conduction studies
**Autoimmune nodopathies**	1. Largely not demyelinating 2. Symmetrical sensorimotor involvement 3. Elevated CSF protein 4. Abnormal nerve conduction studies

**TABLE 3:** Comparative distinguishing features, diagnostic clues, and response to therapy.

Condition	Distinguishing features	Diagnostic clues	Response to therapy
**Guillain–Barré syndrome (GBS)**	1. GBS has an acute onset (< 4 weeks), monophasic, while CIDP has subacute to chronic onset and increases over (> 8 weeks). 2. Cranial nerve involvement is more common in GBS as compared to CIDP 3. GBS is more severe with increased risk of mechanical respiratory failure as compared to CIDP.	1. Diffuse nerve enlargement is seen in CIDP, while in GBS it is less prominent or normal.	1. Corticosteroids are effective in CIDP but not in GBS, and sometimes worsen the outcomes. 2. GBS does not require long‐term therapy like CIDP. 3. Relapse rates are higher in CIDP, requiring maintenance therapy, while they are lower in GBS.
**Multifocal motor neuropathy (MMN)**	1. MMN is pure motor, while CIDP can cause both motor and sensory effects. 2. MMN is asymmetric and CIDP is also asymmetric. 3. The Onset of MMN is usually in the distal upper limb	1. While CSF protein levels might be mildly increased in MMN, they are generally not as high compared to those seen in CIDP (Van Den Bergh et al. 2022). 2. Nerve conduction block without sensory involvement in MMN.	The response to corticosteroids and plasma exchange in MMN is poor, and in some cases ineffective, but not deteriorating (Van Schaik, Léger, et al. 2010).
**Charcot–Marie–Tooth disorders** **(CMTDs)**	1. Earlier onset in life as compared to CIDP. 2. Family history is more commonly associated with hereditary neuropathies, while CIDP is an acquired, immune‐mediated polyneuropathy. 3. Pes cavus, Pes planus, and foot drop are more commonly associated with CMT. 4. CMT Not associated with inflammation	1. Genetic testing is positive for CMTDs. 2. Uniform demyelination on NCS while segmental demyelination in CIDP.	1. There is no evidence that corticosteroids are effective in CMT. Any implication of response is misleading
**POEMS syndrome**	1. Organomegaly and endocrine abnormalities are present in POEMS syndrome. 2. Skin changes like hyperpigmentation, hemangiomas, and clubbing are seen. 3. Ascites and effusion, edema, and papilledema can also be present.	1. Monoclonal M protein test is usually positive in POEMS syndrome. 2. Elevated levels of VEGF 3. Sclerotic bone lesions on x‐rays	1. Requires treatment of plasma cell dyscrasia, e.g., lenalidomide, dexamethasone, or stem cell transplant. 2. Partial corticosteroid responses can be confusing diagnostically and may be seen in POEMS syndrome. However, corticosteroid monotherapy and IVIg are usually ineffective, and definitive therapy requires addressing the underlying plasma cell disorder (Faizan et al. 2020).
**Amyloid neuropathy (AL)**	1. Amyloid neuropathy can be painful, while CIDP does not usually present with pain symptoms. 2. Onset is usually subacute or chronic with weight loss and fatigue. 3. Autonomic dysfunction, like orthostatic hypotension, gastroparesis, and erectile dysfunction, is more prominent in AL neuropathy.	1. Monoclonal proteins (serum/urine M‐protein, κ, or λ light chain) are present. 2. Amyloid deposits in nerve/fat pad/rectal tissue (Congo red +, apple‐green birefringence)	1. Treated with amyloidosis therapy, such as chemotherapy and stem cell transplant.
**Vasculitic neuropathy**	1. Vasculitic Neuropathy is painful 2. It is asymmetric mononeuropathy multiplex 3. Patients with vasculitic neuropathy can frequently develop constitutional symptoms like fever, rash, weight loss, and night sweats.	1. Elevated erythrocyte sedimentation rate (ESR) and C‐reactive protein (CRP) 2. May be positive for ANCA or ANA 3. Nerve biopsy shows necrotizing vasculitis.	1. Responds to steroids and immunosuppressants. 2. IVIg may be effective in some refractory cases.
**Paraneoplastic neuropathy**	1. Rapid or subacute progression 2. Often asymmetric distribution but can be symmetric as well. 3. Associated with malignancy (small cell lung carcinoma or ovarian cancers, breast cancers, etc.)	1. Paraneoplastic antibodies (anti‐Hu, CV2/CRMP5, amphiphysin, etc.) are positive. 2. Malignancy workup is positive	1. Poor IVIg and corticosteroid response unless the underlying malignancy is treated.
**Autoimmune nodopathies**	1. Younger age of onset 2. Tremor and sensory ataxia may be present.	1. Positive for IgG4 anti‐NF155/CNTN1/CASPR1 antibodies. 2. Extremely elevated CSF proteins (> 100 mg/dL) may be present	1. More robust and sustained response to rituximab. 2. Poor or transient response to corticosteroids and IVIG.

### 7.1 Guillain–Barré Syndrome

GBS is an immune‐mediated PNS polyneuropathy that is acute in onset. It can occur in both young and elderly patients with an incidence of 1 in 100,000 individuals (Abbassi and Ambegaonkar 2019; Nazir et al. 2024). Many clinical features of GBS overlap with CIDP, specifically A‐CIDP. GBS has rapid progression leading to peak severity within 4 weeks, unlike CIDP, which progresses slowly and reaches nadir after, but a type of CIDP, A‐CIDP, could reach nadir within 4–8 weeks, leading to a significant risk of misdiagnosis between A‐CIDP and GBS. Symptoms of GBS do not persist over 8 weeks, which could help differentiate these conditions, but some cases of GBS after responding well to initial therapy may worsen, known as TRF‐GBS, and cause further problems in distinguishing the two from each other (Inan et al. 2024; Trojaborg 1998).

Both CIDP and GBS are demyelinating conditions presenting symmetrical weakness with abnormal NCS (Abbassi and Ambegaonkar 2019). Furthermore, sensory symptoms like paresthesia and ataxia with absent reflexes are also common in both conditions. The levels of proteins in CSF are increased in both as well. Having an immune‐mediated etiology, CIDP and GBS both respond well to IVIg and PE (Inan et al. 2024; Rałowska‐Gmoch et al. 2024). Although having many similar features, some differences do exist between them and could help clinicians in making a more accurate diagnosis. The most important difference could be the duration of the disease. GBS is acute in onset, usually monophasic (except TRF‐GBS), and treated with short‐term therapy, whereas CIDP is chronic with a nadir after 8 weeks and usually has a remitting and relapsing course, requiring long‐term maintenance therapy (Inan et al. 2024; Grimm et al. 2019). The nerve ultrasound also revealed that CIDP is associated with significant enlargement of nerves, whereas in GBS, nerve enlargement was mild or nerves were normal (Grimm et al. 2019; Toh et al. 2022).

### 7.2 Multifocal Motor Neuropathy

MMN, also known as multifocal motor neuropathy with conduction block, is a rare, acquired condition characterized by progressive and asymmetric motor weakness with sensory deficits (Hameed and Cascella 2023). The involvement of the upper limbs is more common in MMN than the lower limbs (Hameed and Cascella 2023). Similarities between CIDP and MMN include that both conditions are associated with demyelination, have a progressive disease course, and abnormal NCS; both also respond to IVIG. However, significant differences exist that could aid in more accurate diagnosis (Vlam et al. 2012; Allen, Clarke, et al. 2024). These include CSF studies that show a normal or mild increase in proteins in MMN as compared to CIDP (Hameed and Cascella 2023). There is a presence of anti‐ganglioside auto‐antibodies like Anti‐GM1 IgM in MMN, which are not present in CIDP (Hameed and Cascella 2023). On imaging, like ultrasound or MRI, there is symmetric nerve enlargement in CIDP, whereas asymmetric enlargement is observed in MMN. Another diagnostic test that could prove to be useful is the magnetic fatigue test, which revealed that MMN is associated with use‐dependent conduction block, whereas no such characteristic is observed in CIDP (Lauria et al. 2010). The response of MMN to corticosteroids and PE is suboptimal and may even prove to be deteriorating in some cases (Yeh et al. 2020).

### 7.3 Charcot–Marie–Tooth Disorders

Charcot–Marie–Tooth Disorders (CMTDs), also known as hereditary motor and sensory neuropathies (HMSN), are a group of neuromuscular disorders characterized by a progressive or even stationary course, with distal upper and lower limb muscle weakness and wasting as the main symptoms (Kamińska and Kochański 2024). Multiple features are common among CIDP and CMTDs; both are demyelinating conditions affecting both sensory and motor nerves, with a progressive course and respond to IVIg (Miki et al. 2013). The CMTDs have an earlier onset in life, usually with a positive family history and commonly present with pes cavus, whereas CIDP is not hereditary but an acquired condition presenting later in life (Pareyson and Marchesi 2009; Di Sarno et al. 2024). Electrophysiological studies of CMTDs show diffuse and homogenous nerve conduction slowing, usually without a block, and CIDP shows inhomogeneous nerve‐conduction velocity slowing, and often partial motor conduction block. The response to corticosteroids by CMTDs has limited evidence showing no significant improvement in the condition of the disease (Kamińska and Kochański 2024).

### 7.4 POEMS Syndrome

POEMS Syndrome (Polyneuropathy, Organomegaly, Endocrinopathy, M‐Protein, Skin changes) is a set of rare paraneoplastic conditions related to multiple systems caused by plasma cell disorders (S. Yu et al. 2025). The CIDP can mimic neuropathy in POEMS syndrome and can be misdiagnosed as one. The features that are common amongst the two conditions are that they are demyelinating conditions that are progressive in nature, with symmetrical distribution and increased CSF protein levels (Nasu et al. 2012; Brown and Ginsberg 2019; Ji et al. 2012). Electrophysiological can also be similar in two conditions, and these similarities have led to misdiagnosis in multiple cases in the past (Nasu et al. 2012). However, some differences are worth mentioning that could help clinicians better understand the two conditions and diagnose them more accurately. These differences include involvement of multiple systems in POEMS syndrome and presence of neuropathic pain in POEMS syndrome, particularly in the lower extremities, like neuropathic foot, which is usually absent in CIDP (Nasu et al. 2012; Wang et al. 2019). Imaging reveals that in POEMS syndrome, there is significant axonal damage with endoneural neovascularization, while CIDP has greater endoneural inflammation and onion‐bulb formation (Nasu et al. 2012; Piccione et al. 2016). Electrophysiologically, the terminal latency index (TLI) was higher in patients with POEMS than in CIDP (Guo et al. 2014). Patients with POEMS demonstrated a higher frequency of absent CMAP, less conduction block, and less temporal dispersion than the CIDP group (Wang et al. 2019). There are also increased levels of vascular endothelial growth factor (VEGF), monoclonal M protein in POEMS syndrome, and there could be presence of sclerotic bony lesions on x‐rays as well (Wang et al. 2019; Dispenzieri 2005). POEMS syndrome does not respond well to corticosteroid monotherapy or IVIg, unlike CIDP (Brown and Ginsberg 2019). It could, however, be treated with lenalidomide, stem cell therapy (Royer et al. 2013).

### 7.5 Amyloid Neuropathy

Amyloidosis is characterized by the deposition of misfolded β‐pleated sheets in different tissues (Bustamante and Zaidi 2023). Depending on the type of precursor amyloid protein, amyloidosis can be divided into different types, including immunoglobulin amyloid light chain (AL), amyloid A (AA), and amyloid Transthyretin (ATTR) (Shin and Robinson‐Papp 2012). Deposition of amyloid proteins in nerves can cause a rare type of neuropathy called amyloid neuropathy (Shin and Robinson‐Papp 2012; Qian et al. 2021). The overlapping features of amyloid neuropathy and CIDP include symmetrical distribution of neuropathy, progressive course of disease, involvement of both sensory and motor nerves, and elevated levels of proteins in CSF (Shin and Robinson‐Papp 2012; Gertz 2022). The key differences between the two conditions are that amyloid neuropathy can present with neuropathic pain and multisystem involvement with weight loss and fatigue, which are usually not seen in CIDP (Shin and Robinson‐Papp 2012; Koike et al. 2021). In amyloid neuropathy, biopsy reveals amyloid aggregates in nerves and apple green birefringence on Congo red staining, and there is the presence of monoclonal proteins (serum/urine M‐protein, κ or λ light chain) (Mathis et al. 2012; Briani et al. 2023). Autonomic symptoms are hallmarks of amyloid neuropathy, such as orthostatic hypotension, gastroparesis, and erectile dysfunction, which are rare in CIDP (Adams et al. 2021). Amyloid neuropathy is treated with chemotherapy based on bortezomib or melphalan and dexamethasone or daratumumab and stem cell therapy (Namiranian and Geisler 2022; Gertz 2018).

### 7.6 Vasculitic Neuropathy

Vasculitides are a group of systemic or localized disorders characterized by damage to blood vessels resulting from the infiltration of inflammatory cells (Gwathmey et al. 2014). This inflammatory process could also involve peripheral nerves; about 60%–70% of systemic vasculitis disorders have been associated with neuropathy (Gwathmey et al. 2014). Common features amongst CIDP and vasculitic neuropathy include having an inflammatory etiology, abnormal NCS, can involve peripheral nerves, and both sensory and motor deficits, with absent or reduced reflexes (Becker et al. 2024; Koike et al. 2022; James et al. 2018). However, vasculitic neuropathy is painful and presents as mononeuropathy multiplex, characterized by asymmetrical distribution, whereas CIDP is a polyneuropathy with a symmetric distribution and is usually painless (Gwathmey et al. 2014). Patients with vasculitic neuropathy can also present with constitutional symptoms like fever, fatigue, weight loss, and night sweats (Gwathmey et al. 2014; Samson et al. 2014). The laboratory findings reveal increased ESR, CRP in vasculitic neuropathy, while they are not raised in CIDP. Furthermore, some types of vasculitic neuropathies can be positive for ANA and ANCA (Gwathmey et al. 2014). Biopsy in vasculitic neuropathy can reveal necrotizing vasculitis and fibrinoid necrosis (Gwathmey et al. 2014). Both CIDP and vasculitic neuropathy can be treated with corticosteroids and immunosuppressants. IVIg is also used in refractory cases (Blaes 2015).

### 7.7 Paraneoplastic Neuropathy

Paraneoplastic neuropathy is a condition that is caused by antibodies against the neural that are generated by the immune system in response to a tumor or malignancy. Among these, anti‐Hu (ANNA‐1), anti‐CV2 (CRMP5), and anti‐amphiphysin antibodies are well characterized, along with many other less common antibodies (Camdessanché et al. 2002; Psimaras et al. 2010). The overlapping features between CIDP and paraneoplastic neuropathy include PNS involvement, damage to both sensory and motor nerves, reduced or absent reflexes, demyelination, and abnormal conduction studies (Camdessanché et al. 2002). Other similarities include a progressive course of disease and elevated levels of protein in CSF (Psimaras et al. 2010; Tian et al. 2022; Jitprapaikulsan et al. 2021). While paraneoplastic conditions are associated with malignancies with their own set of symptoms, sometimes the initial presentation is neuropathy, which can cause problems in diagnosing the condition accurately and can lead to incorrect diagnosis of CIDP or vice versa. The differences that could help differentiate these conditions become even more important as delay in treatment due to initial misdiagnosis can lead to delay in treatment, which could have severe implications for the health of the patient. These paraneoplastic neuropathies have a rapid or subacute progressive nature, usually having asymmetric distribution. They have paraneoplastic antibodies in the serum, and the workup for malignancy is positive (Camdessanché et al. 2002; Jitprapaikulsan et al. 2021). They do not respond much to IVIg, corticosteroids, or immunosuppression unless the underlying malignancy is treated (Camdessanché et al. 2002; Keime‐Guibert et al. 2000).

### 7.8 Autoimmune Nodopathies

Autoimmune nodopathies were previously classified as a type of CIDP but are now a set of distinct clinical entities (Pascual‐Goñi et al. 2021). This change was brought about recently after discovery of nodal and paranodal antibodies such as neurofascin‐155 (NF‐155), Caspr‐1 and contactin‐1 (CNTN‐1) in patients with immune‐mediated neuropathy (Broers et al. 2024; Khadilkar et al. 2022). Most of the clinical features of this disorder fulfill the criteria for CIDP, but the response to treatment varies, requiring the establishment of a more rigorous diagnostic criterion to be followed to avoid misdiagnosis (Broers et al. 2024; Zhao et al. 2024). Both CIDP and autoimmune nodopathies have an autoimmune etiology, demyelination of nerves, involvement of both sensory and motor nerves, elevated level of proteins in CSF, and abnormal conduction studies (Pascual‐Goñi et al. 2021; Broers et al. 2024). The autoimmune nodopathies can be differentiated from CIDP by earlier onset of disease at a younger age, and presence of tremors and sensory ataxia (Broers et al. 2024; Khadilkar et al. 2022). There is a presence of IgG4 antibodies like NF155, CNTN1, and CASPR1, and CSF proteins may be extremely elevated, greater than 100 mg/dL, in autoimmune nodopathies (Kira 2021). Autoimmune nodopathies do not respond well to IVIg but show a more sustained and robust response to rituximab (Martín‐Aguilar et al. 2022).

## 8 Conventional Therapy for CIDP

### 8.1 Corticosteroids

Corticosteroids are one of the most important and widely used classes of anti‐inflammatory drugs in the world (Schacke 2002). Corticosteroids reduce the inflammatory processes in CIDP by several different mechanisms, including reducing the nuclear factor‐kB (NF‐kB), reducing the activity of phospholipase A2, and reducing the production of different inflammatory mediators, eicosanoids, and cytokines. (Rhen and Cidlowski 2005)

Studies suggest that corticosteroid use has led to a better prognosis for CIDP. Studies also revealed that corticosteroids led to remission of CIDP in patients. Patients receiving corticosteroids had improvement in score in the seven‐point GBS disability grade scale, RMI, and INCAT disability scale (Hughes et al. 2017; van Schaik, Eftimov, et al. 2010). They had an improvement in muscle strength on average 6 months period (Hughes et al. 2017).

Another clinical trial comparing the pulsed high‐dose dexamethasone and standard prednisolone treatment for CIDP revealed that remission was 40% higher than baseline in both treatments. After a year, 16 out of 40 patients were in remission, 10 out of 24 in the dexamethasone group, and six out of 16 in the prednisolone group. Both these treatments were equally effective for CIDP (van Schaik, Eftimov, et al. 2010).

Although corticosteroid use has been used as standard therapy for CIDP, long‐term and sustained use of these drugs has been associated with a wide variety of adverse events. Anti‐inflammatory and immunosuppressive actions can lead to infections and increase the need for antibiotic usage (Schacke 2002). The effect of corticosteroids on carbohydrate metabolism can lead to hyperglycemia and diabetes mellitus. Their mineralocorticoid effects can increase salt retention, and potentiation of angiotensin 2 can lead to hypertension; it has also been associated with osteoporosis in adults (Rhen and Cidlowski 2005; Hughes et al. 2017). Other side effects included weight gain, hirsutism, moon facies, sleeplessness, cataract, peptic ulcer, gastrointestinal hemorrhage, and psychiatric manifestations (Hughes et al. 2017; Heffler et al. 2018).

### 8.2 Intravenous Immunoglobulin

IVIg is an anti‐inflammatory and immunomodulatory agent when given at high doses (Lünemann et al. 2015). IVIg is extracted from blood and plasma pooled from 10,000 people and consists mainly (> 95%) of IgG. Since it is collected from a large population, it contains a large variety of antibodies that are directed to various self and foreign antigens (Lünemann et al. 2015). IVIg protects the Schwann cells by blocking the Fc receptor on macrophages, preventing antibody‐mediated attack (Hughes et al. 2014). IVIg upregulates the FCγR2B receptor, which is a low‐affinity inhibitory receptor that increases the threshold for activation of B‐lymphocytes (Tackenberg et al. 2009). It suppresses the activation of the complement cascade and formation of the membrane attack complex (Dalakas 2002; Basta et al. 1996). It reduces the production of inflammatory cytokines, chemokines, and adhesion molecules (Dalakas 2004). IVIg also decreases the B‐cell activating factor on B‐lymphocytes and monocytes (Bick et al. 2013). Normal IgG, after taken up by epithelial cells through pinocytosis, is protected from catabolism by binding with FcRN. Still, when there is a large number of exogenous immunoglobulins present, it overdrives this recycling mechanism and causes increased degradation of autoantibodies by lysosomes (Z. Yu and Lennon 1999).

The usual induction dose of 2 g/kg for 2–5 days and is followed by maintenance dosing of 1 g/kg every third week (Layton et al. 2023). IVIg is equally effective as corticosteroids in treating CIDP patients; however, there were some subtle differences in some patients. IVIg has been shown to have a better response depending on the variant of CIDP (Querol and Lleixà 2021). In a clinical trial, it has been observed that 0.5 and 2.0 g/kg maintenance dosing of IVIg has improved INCAT score by 65% and 92% respectively. Responder rates for grip strength, I‐Rod and MRC sum were 56%, 38%, and 59% respectively for 0.5 g/kg dosage, and 83%, 72%, and 86% respectively for 2.0 g/kg dosage (Cornblath et al. 2022). In another study, patients were given IVIg and 10% caprylate/ chromatography purified (IGIV‐C), loading dose was 2 g/kg and then maintenance dose of 1 g/kg for every 3 weeks was given, showed significant improvement in INCAT score; 47% patients by third week and 53% patients after second infusion at sixth week, these patients continued to improve over the 24 weeks trial period.

Adverse events related to IVIg usage were minor and occurred in a minority of patients (Lünemann et al. 2015). One of the major side effects associated with IVIg use was headache and nausea (Allen et al. 2020). Other minor adverse events included pyrexia, chills, myalgia, back pain, and chest discomfort (Dalakas 2004; Cornblath et al. 2023). These events could be held under control by using corticosteroids and slowing down the rate of infusion. Some serious but rare events were thromboembolic events, including stroke, myocardial infarction, and pulmonary embolism (Dalakas 1997). These events occurred due to increased viscosity due to IVIg infusion and were more common in patients with more advanced age, immobilized patients, patients with cardiovascular problems, and history of thrombotic events (Allen et al. 2020). Other side effects included acute tubular necrosis in patients with pre‐existing kidney disease, severe anaphylactic reaction in patients with IgA deficiency, aseptic meningitis in patients with migraine, and dermatological conditions (Dalakas 2004; Cornblath et al. 2022; Allen et al. 2020).

### 8.3 Subcutaneous Immunoglobulin

IgG therapy is a well‐recognized long‐term remedy for CIDP, typically given intravenously (IVIg). The SCIg delivery method is a secure and efficient alternative that is authorized by the United States Food and Drug Administration (FDA) and is currently in wide use for the treatment of adults with CIDP. Many articles (Van Den Bergh et al. 2022; Gorson 2012) talked about the efficacy of both immunoglobulin delivery routes, and results showed that IVIg and SCIg exhibit similar long‐term effectiveness in CIDP. Nevertheless, SCIg may offer extra advantages for certain patients, such as eliminating the need for venous access or premedication and decreasing the incidence of systemic side effects. Local‐site reactions occur more frequently with SCIg compared to IVIg, but they are generally well‐tolerated and diminish with following infusions. Evidence indicates that numerous patients favor SCIg after switching from IVIg (Van Den Bergh et al. 2022).

### 8.4 Plasma Exchange (Plasmapheresis)

Plasmapheresis or therapeutic plasma exchange (TPE) is a procedure in which blood is extracted from the body and plasma is separated from blood by filtration or centrifugation, and this plasma is then discarded. Meanwhile, a substitute fluid (albumin, fresh frozen plasma, cryosupernatant, Ringer's lactate or normal saline) is reinfused in the body (El‐Ghariani and Unsworth 2006; Nakanishi et al. 2014; Mehndiratta et al. 2015; Madore 2002). The principle of plasmapheresis is to remove all the antibodies, cytokines, chemokines, components of the complement system, and other inflammatory mediators from plasma to reduce the damage caused by these on the neurons and other cells (Stino et al. 2021). TPE is reserved for emergencies, severely disabled patients, and cases that are refractory to other treatments like corticosteroids and IVIg (El‐Ghariani and Unsworth 2006; Stino et al. 2021). PE is equally effective as IVIg and corticosteroid in treating CIDP and can be used as an alternative therapy for patients if they are having adverse effects to IVIg or corticosteroids (Stino et al. 2021).

One of the major factors leading to not using TPE are need for specialized staff, continuous monitoring for adverse effects and transient beneficial effects, after which 50%–67% of cases start to relapse within weeks to months (Stino et al. 2021; Schröder et al. 2009). Adverse events related to TPE can be divided into two major subgroups: Either due to a problem in vascular access or a reaction to substances in the substitution fluid. Adverse effects related to vascular access are infections related to catheter use, hematomas, and pneumothorax, while components of substitution fluid can result in anaphylactoid reactions, thrombosis, and hypocalcemia due to citrate (Madore 2002; Mehndiratta et al. 2015). Acute complications of PE include electrolyte imbalance, hypotension due to vasovagal reaction, cardiac arrhythmia, and chronic use of PE can lead to iron deficiency, hemolysis, and immunosuppression due to hypogammaglobulinemia (Mehndiratta et al. 2015; McMillan et al. 2013).

One sham‐controlled trial revealed that PE led to significant improvements in different neurologic disability scales related to weakness, reflexes, and nerve conduction (Dyck et al. 1986). Another study that was conducted between 2012 and 2015 comparing PE and immunoadsorption (IA) revealed that PE had significant improvements in INCAT and MRC score, although less than IA (Lieker et al. 2017).

### 8.5 Monoclonal Antibodies

Monoclonal antibodies are like IVIg as they imply the use of antibodies to reduce the inflammatory process in CIDP, but monoclonal antibodies are specific for a single antigen and provide more targeted effects as compared to IVIg (Nelson et al. 2000). There are a few monoclonal antibodies that have been hypothesized to be used for CIDP, including rituximab, alemtuzumab, and daratumumab. All of these have different mechanisms, but they all tend to modulate the influence of the immune system on the nervous system. Rituximab binds to the CD‐20 receptor found on B‐cells (Hu et al. 2022). Alemtuzumab is a recombinant humanized (IgG1Κ) monoclonal anti‐CD‐52 antibody (Brannagan and Patterson 2010). Daratumumab is an anti‐CD38 antibody; CD38 antigens are present on plasma cells (Scheibe et al. 2022).

These antibodies have been shown to have variable efficacy in treating CIDP, but these data have been obtained only from some small‐scale studies. Rituximab in a meta‐analysis revealed MRC improvement of 1.3 (95% CI −2.6 to −0.1, *p* value = 0.04) and INCAT improvement of 1.7 (95% CI 1.0–2.3, *p* value < 0.0001), but this study was performed before autoimmune nodopathies were classified separately from CIDP (Hu et al. 2022). However, in the latest study by Orazio et al., no significant difference was found between rituximab and placebo at 12 and 18 months, and CIDP worsened in both groups (Nobile‐Orazio et al. 2024). Another open‐label trial by Doneddu et al., revealed rituximab to be safer and effective in patients with CIDP not responding to conventional treatment (Doneddu et al. 2024). Furthermore, a trial investigating low‐dose rituximab use for long‐term also revealed it to be safer and effective (Zheng et al. 2024). These variable findings warrant further investigation of rituximab as a possible therapy for CIDP.

A study using alemtuzumab in CIDP, which consisted of 7 patients, had revealed complete remission in 2 patients, partial remission in 2 patients, and no improvement in the remaining 3 patients (Marsh et al. 2010). Daratumumab also resulted in improvement of the INCAT score in a clinical study (Scheibe et al. 2022).

Side effects regarding alemtuzumab include secondary autoimmunity, B‐cell clones that form antibodies against self‐antigens become activated and lead to autoimmune diseases such as autoimmune thyroiditis and autoimmune hemolytic disease (van der Zwan et al. 2020). These autoimmune diseases were more common in patients with higher IL‐21 levels and could be avoided if IL‐21 levels are checked before alemtuzumab treatment (Brannagan and Patterson 2010). Most of the adverse events related to rituximab were infusion‐related, which could include hypersensitivity, fever, rash, nausea, and cytokine release syndrome (Vital et al. 2000). All these monoclonal antibodies could lead to immunosuppression and infections (Scheibe et al. 2022; Marsh et al. 2010; Paul and Cartron 2019).

### 8.6 Immunosuppressive Agents

Immunosuppressive agents are considered in the treatment of CIDP when first‐line treatments, corticosteroids, IVIg, and PE, are contraindicated or ineffective, not well tolerated, or require long‐term use and become cumbersome or predispose to adverse effects. Such agents are added as an adjunct or steroid/IVIg‐sparing therapy and are supposed to be given long enough before measuring action efficacy. One of these drugs is azathioprine, which has received widespread clinical application, though a randomized trial of 9 months failed to demonstrate additional benefit compared to prednisone alone; nonetheless, longer‐term outcomes suggest that it contributes to the maintenance of remission and the elimination of steroid dependence (Gorson 2012).

Cyclosporine A has shown high response rates of 40%–90% in small series of refractory patients, such that steroids have been withdrawn in some patients. Cyclophosphamide, in pulse intravenous form, has proven useful even in patients who are unresponsive to IVIg, PE, or steroids, with 80% response reported and an average time to respond of 8.5 months (Gorson 2012). Mycophenolate mofetil has demonstrated a good response in corticosteroid reduction within 34 months of therapy onset. Interferon alpha 2a and interferon beta‐1a were among the first biologics to show promise in uncontrolled trials; however, a subsequent large trial of interferon beta‐1a, in a randomized, controlled design, did not confirm its clinical efficacy. On the same note, methotrexate, which is effective in inflammatory myopathies, was found not to be superior to placebo in a controlled trial; still, the limitations of the trial left its place to be undetermined (Gorson 2012).

Other immunosuppressants like etanercept, tacrolimus, and hematopoietic stem cell transplantation have been discussed in a small number of reports, and although they may have an application in treatment‐refractory patients, they are not routinely used due to safety or data concerns (Gorson 2012) (Table 4).

**TABLE 4:** Treatment regime.

Treatment	Mechanism of action	Efficacy	Administration	Side effects	Notes
Corticosteroids	Suppress the immune response and inflammation (e.g., dexamethasone, prednisolone)	Proven effective against placebo, similar to IVIg	Oral or intravenous (IV)	Diabetes Mellitus, Hypertension, Osteoporosis, Infections, sleeplessness, cataract, moon face, peptic ulcer	Long‐term use can lead to significant side effects but offers similar efficacy to IVIg.
IVIg (Intravenous immunoglobulin)	A collection of antibodies that modulate immune function	Proven effective against placebo, often first‐line	Intravenous (IV)	Headache, nausea, thromboembolic events, pyrexia, chills, myalgia, back pain, chest discomfort	Generally well‐tolerated, widely used as a first‐line treatment.
Plasma exchange (PE)	Removes antibodies and other immune components causing inflammation in CIDP	Effective but typically transient effects relapse in months or weeks	Intravenous (IV)	Hypotension, anaphylactoid reactions, infections, hematomas, pneumothorax, thrombosis, hypocalcemia	Requires frequent sessions; usually used in severe cases or refractory cases.
SCIg (Subcutaneous immunoglobulin)	Delivers IgG subcutaneously; immune modulation similar to IVIG	Comparable to IVIg in long‐term efficacy	Subcutaneous (SC)	Local injection site reactions (mild), rare systemic effects	FDA‐approved; preferred by some patients due to fewer systemic effects and home use convenience.
Monoclonal antibodies	Antibodies that target specific immune system components (e.g., alemtuzumab, Rituximab, daratumumab)	Emerging evidence, effectiveness has been proved in small‐scale studies	Intravenous (IV) or subcutaneous (SC)	Increased risk of infection, infusion reactions, autoimmunity, hypersensitivity, fever, rash, nausea, cytokine release syndrome	Rituximab and other monoclonal antibodies are increasingly explored, especially in refractory cases.
Immunosuppressive AGENTS	Inhibit immune cell proliferation (e.g., azathioprine, cyclophosphamide, cyclosporine)	Variable efficacy; useful in refractory or steroid‐sparing settings	Oral or IV (depending on agent)	Myelosuppression, hepatotoxicity, infections, GI upset, malignancy (long‐term)	Used when first‐line therapies fail or are not tolerated; includes azathioprine, cyclosporine, cyclophosphamide, mycophenolate mofetil, methotrexate.

#### 8.6.1 Efgartigimod: A New Horizon in CIDP Treatment

Limited efficacy and significant side effects of immunomodulatory therapies, such as corticosteroids and IVIg, have spurred the search for more targeted and effective treatments. Efgartigimod, a novel therapeutic agent, has emerged as a potentially effective option for managing chronic inflammatory demyelinating polyradiculoneuropathy (CIDP).

#### 8.6.2 FcRn Mechanism of Action

Efgartigimod, or ARGX‐113, is a humanized IgG1‐derived crystallizable (Fc) region that competitively binds the FcRn (Goebeler et al. 2022; Vanoli and Mantegazza 2022). It has ABDEG mutations that increase its affinity for the IgG‐binding site of FcRn. When IgG is endocytosed in a mildly acidic environment of the endosome, the Fc fragment of IgG, which is a ligand for FcRn, binds to it and prevents its lysosomal degradation (Goebeler et al. 2022; Blair 2024). Efgartigimod competes with the IgG antibodies for binding to FcRn. This reduces the circulating IgG antibodies, including pathogenic and autoantibodies, without affecting other immunoglobulins and albumin. This reduction in circulating IgG may be therapeutically relevant in CIDP, particularly in patients in whom pathogenic IgG autoantibodies contribute to disease mechanisms (Vanoli and Mantegazza 2022; Howard et al. 2023).

Efgartigimod is a FcRn blocker derived from human immunoglobulin G1 (IgG1) and produced in Chinese hamster ovary cells. The efgartigimod alpha Fc fragment is a homodimer composed of two identical peptide chains. Each protein consists of 227 amino acids joined by two interchain disulfide bridges and has an affinity for the FcRn (FDA and CDER 2024).

#### 8.6.3 Pharmacokinetic Data on Efgartigimod

The recommended dosage of efgartigimod is 1008 mg, administered subcutaneously over approximately 30–90 s as a once‐weekly injection. Efgartigimod is given in combination with hyaluronidase, an endoglycosidase, that enhances permeability of the subcutaneous tissue by depolymerizing hyaluronan. The suggested dosage is 1008 mg /11,200 units of hyaluronidase (FDA and CDER 2024).

#### 8.6.4 Results of the ADHERE Study

The ADHERE study (Allen, Basta, et al. 2024) showed that efgartigimod considerably decreased the likelihood of clinical deterioration as compared to placebo. It also results in significantly better scores on the Inflammatory Rasch‐built Overall Disability Scale(I‐ROD) and increased grip strength. The drug was well tolerated, with mild‐to‐moderate adverse effects. Patients first entered a run‐in period in which previous medication was discontinued, followed by required clinical worsening and participation in stage A, during which the medicine would be supplied open‐label. If improvement occurred, patients advanced to stage B and were then randomly assigned to receive the medication or a placebo for up to 48 weeks. The primary outcome was the proportion of relapses that occurred in each group. The primary endpoint was met with efgartigimod, considerably reducing the incidence of CIDP relapse in stage B by 61%. This represents a relative risk reduction; the absolute difference in relapse rates between groups was more modest, underscoring the importance of interpreting these findings in both relative and absolute terms. A 78% improvement rate among subjects who received at least 4 injections of the active drug to achieve a full IgG‐lowering effect was also observed.

The ADHERE study was conducted on patients aged between 18 and 85 years. The clinical indications for patients in this trial were, if they have definite or probable CIDP, a CIDP Disease Activity Status (CDAS) score of at least 2 at screening, and an Inflammatory Neuropathy Cause and Treatment (INCAT) score of at least 2 (a score of 2 had to be exclusively from leg disability) at the first run‐in visit or stage A baseline. Patients who had not received treatment for 6 months were included, and those who were receiving treatment within the past 6 months or had recently stopped treatment had to demonstrate evidence of clinically meaningful deterioration (ECMD) in the run‐in period to be included in the trial. They did not include the patients who had low levels of IgG because efgartigimod reduces IgG, and patients who are IgG deficient will have greater chances of infection. Also, the patients with polyneuropathy from different causes and the pure sensory form of CIDP were excluded from the study (Allen, Basta, et al. 2024). Importantly, the ADHERE trial did not specifically enroll or analyze a refractory CIDP population; therefore, conclusions regarding efficacy in treatment‐resistant patients cannot be drawn.

Efgartigimod was approved by the FDA in December 2021 to treat generalized myasthenia gravis (gMG) while also being studied for the treatment of other autoimmune diseases, such as pemphigus, autoimmune myositis, immune thrombocytopenia, bullous pemphigoid, and chronic inflammatory demyelinating polyradiculoneuropathy (FDA and CDER 2024). With its FDA approval in June 2024, efgartigimod now offers a new approach to managing CIDP with reduced dependence on Immunosuppressants and a more targeted approach (Heo 2022).

#### 8.6.5 Safety

Efgartigimod is generally well tolerated with a relatively low risk of adverse events, with most being mild to moderate reactions (Allen, Basta, et al. 2024). The most common adverse events included headache, nasopharyngitis, diarrhea, nausea, and upper respiratory tract infections (Vanoli and Mantegazza 2022; Howard et al. 2021, 2023). Other adverse events included infections and infusion site reactions, like infusion site erythema and injection site bruising. Few deaths were reported in the clinical trials, though none were deemed related to efgartigimod by the researchers (Vanoli and Mantegazza 2022; Blair 2024; Howard et al. 2021).

#### 8.6.6 Current Knowledge Gaps

While corticosteroids have a broad immunosuppressive effect and are not specific to IgG reduction. Efgartigimod primarily inhibits Fc receptor and reduces IgG, proving as a more targeted and effective therapy. Generally, corticosteroids have a slower onset of action compared to efgartigimod, which showed improvements typically observed 1–2 weeks after the first infusion (Bril et al. 2024). Furthermore, corticosteroids are known for more significant side effects, especially with long‐term use, while efgartigimod is well‐tolerated with mostly mild adverse events.

Efgartigimod has been shown to be comparable to IVIg in reducing immunoglobulin levels and improving symptom scores in patients with myasthenia gravis (Tran 2022). It may be considered as an alternative for patients on long‐term IVIg regimens. While IVIg provides a pool of normal antibodies to modulate the immune response, efgartigimod is more targeted. Further studies are needed to fully understand the efficacy and long‐term effects of efgartigimod compared to IVIg.

Importantly, not all FcRn inhibitors have demonstrated efficacy in CIDP. A randomized trial of rozanolixizumab in CIDP did not meet its primary endpoint, suggesting that FcRn inhibition may not uniformly translate into clinical benefit across agents and underscoring the need for cautious interpretation of class effects (Querol et al. 2024).

Where elimination of antibodies other than IgG is required, or simultaneous replacement of absent plasma proteins is required for therapeutic effect, PE is favored over FcRn inhibition (Mina‐Osorio et al. 2024). However, compared with other immunoglobulins, FcRn inhibition is associated with less invasive access requirements, more targeted elimination of IgG without significantly affecting circulating protein levels, and limited effects on other therapeutic medication levels relative to other monoclonal antibodies (Ulrichts et al. 2018).

Since anti‐FcRn monoclonal antibodies (mAbs) and fragments have just recently entered this field, there is a dearth of information on their long‐term safety and efficacy. As of yet, FcRn targeting has not been investigated for use in acute flare‐ups of IgG‐mediated illnesses or crisis situations. Although IVIg, immunoglobulin, corticosteroids, and PE are effective in treating CIDP, certain patients do not respond to any of these treatments. While efgartigimod expands the available treatment options, its efficacy specifically in treatment‐refractory populations has not yet been established and requires further investigation (Stino et al. 2021).

Several other novel treatments, such as SAR445088 (Chow et al. 2023), which is a complement pathway inhibitor, and bruton tyrosine kinase (BTK) inhibitors (Ringheim et al. 2021), which inhibit B cell maturation have been developed recently. As research progresses and clinical trials move into later phases, we may see these agents emerge as valuable additions to the therapeutic arsenal for CIDP patients. Future directions include exploring combination therapies with other immunomodulatory drugs, investigating their efficacy in different CIDP subtypes, and evaluating their long‐term safety and tolerability.

However, CIDP is a heterogeneous condition, with significant rates of treatment‐free remission on current medications and a wide range of therapeutic responses from patient to patient. The results of previous and ongoing investigations point to the need for a bigger treatment arsenal for patients with CIDP, but further investigation is required to pinpoint the precise role and worth of each drug.

## 9 Conclusion

To sum up, CIDP is a complicated immune‐mediated neuropathy, with heterogeneous clinical manifestations, common features of clinical presentation with other neuropathic conditions, and inconsistent response to treatment. The current progress in knowledge of its immunopathogenesis, such as the contributions made by cellular immunity, humoral processes, and the loss of the blood–nerve and blood–brain barrier, have enhanced the accuracy of diagnosis and therapeutic approach. Traditional therapies with corticosteroids, IVIg, and PE continue to be the mainstay of intervention, but constraints such as adverse effects, relapse, and treatment resistance underscore the need to have more specific treatment. Newer therapies, especially FcRn inhibitors like efgartigimod, are also on the horizon and are particularly encouraging, as they target pathogenic IgG levels and have shown promising clinical results in recent trials. Further scientific efforts are necessary to optimize diagnostic criteria and to establish credible biomarkers and define the long‐term effectiveness and the best place of new treatments in CIDP management algorithms, with the final goal of improving patient outcome and quality of life.

## Author Contributions

**Ayesha Khan**: conceptualization, investigation, writing – original draft, visualization, methodology, software, formal analysis, data curation. **Arsal Khan**: conceptualization, writing – original draft, methodology, visualization, software, formal analysis, data curation, resources. **Kuldeep Dalpat Rai**: investigation, writing – original draft, validation, visualization, methodology, software, data curation, resources. **Anzel Saeed**: writing – original draft, investigation, methodology, visualization, software, resources. **Harsh Kumar**: investigation, writing – original draft, methodology, validation, software, formal analysis, data curation. **Aneesh Kumar Sangtiani**: investigation, writing – review and editing, validation, methodology, formal analysis, software, data curation. **Tehreem Fatima**: investigation, validation, visualization, writing – review and editing, formal analysis, software, data curation. **Muhammad Tanveer Alam**: investigation, validation, methodology, formal analysis, project administration, data curation, resources. **Faiza Rajput**: investigation, methodology, visualization, writing – review and editing, software, project administration, data curation. **Sakshi Chawla**: investigation, validation, writing – review and editing, methodology, formal analysis, software, data curation. **Hussain Haider Shah**: writing – review and editing, validation, methodology, software, project administration, investigation, writing – original draft, data curation. **Humaira Kalam**: writing – review and editing, methodology, visualization, software, formal analysis, supervision, resources, funding acquisition.

## Funding

The authors have nothing to report.

## Ethics Statement

The authors have nothing to report.

## Consent

The authors have nothing to report.

## Conflicts of Interest

The authors declare no conflicts of interest.

## Data Availability

The data that support the findings of this study are available from the corresponding author upon reasonable request.
